# New Drugs, Appliances, and Things Medical

**Published:** 1891-10-03

**Authors:** 


### NEW DRUGS, APPLIANCES, AND THINGS MEDICAL.

[All preparations, appliances, novelties, etc., of which a notice is desired, should be sent for The Editor, to care of The Manager, 140, Strand, London, W.C.]

### AT THE BRITISH MEDICAL ASSOCIATION.

(*Continued.*)

Messrs. Idris and Co., Kentish Town, had a first-rate exhibit of their mineral waters, fruit cordials—we have mentioned these before as most excellent for effervescing drinks—lime juice, and the vehicles for effervescing water, viz., syphons and gasogen es.

Amongst the instrument makers' exhibits, that of Messrs. Mayer and Meltzer made a brave show. All kinds of standard instruments were to be seen here, and the latest modification or invention. Mr. A. E. Barker's irrigating scoop, by means of which a sinus or abscess cavity can be well scraped, and at the same time the scrapings washed out by a constant flow of an antiseptic fluid, or of plain boiled water; gynæcological instruments of never-ending variety; throat, ear, and eye instruments, the results of the inventive skill of the foremost men in these departments of practice; an excellent collection of anæsthetic apparatus, including the late Dr. Sheppard's very useful angle mount for cases where the patient has to lie with the face downwards.

Messrs. Lynch and Co., Aldersgate Street, London, had an interesting table of exhibits. Amongst them we noticed an excellent series of aluminium instruments. The advantages claimed for these are: (*a*) Extreme lightness and cheapness; (*b*) no replating required; (*c*) not acted on by the usual reagents. They include uterine instruments, urethral vaginal, ear, nasal, and rectal specula, probes of all kinds, dressing instruments, and others of every-day use. The table also held quite a shopful of well made surgeon's tools of all sorts.

Messrs. Down Bros., 5 and 7, St. Thomas's Street, S.E., showed a number of surgical instruments especially constructed to be aseptic. Thus, amputation knives, bistouries, scalpels, chisels, and gouges, and even instrument cases, were exhibited, all forged out of the solid, so that there should be no crevices to hold germs as may happen where the handles are made of wood or ivory and rivetted to the blade. They had a good collection of laryngeal instruments of all kinds. Among them we noticed Dr. Hall White's recently-invented tongue depressor, with glass shield, for examination of the throat in infectious cases. This prevents the expectoration of the patient being blown into the doctor's face and mouth, and so possibly inflicting him with diphtheria, syphilis, or the like. Among the nasal instruments we noticed Krause's Snare, as recommended by Mr. Charters Symonds, and the very useful self-retaining specula of Neil Griffith. A very complete set of abdominal operation instrument next attracted our attention; there were Makin's bowel clamps to hold the bowel, fine needles to put Lembert's suture into it, Lenn's bone plates to facilitate its apposition and many other ingenious inventions by which modern surgeons are enabled to cut, join, and mend almost any part of the abdominal tube. Next an excellent binaural stethoscope was shown us; this had a clamp, so that when fixed to the ears and the right pressure applied, it was maintained in that position. Cathcart's steriliser for surgical instruments came under notice, to be followed by a fine array of glass trays for disinfecting instruments, carbolising catheters, and for dressing wounds of any part of the body. These with aseptic ligatures in great variety and a useful chloroform drop-bottle with space in the cap for nitrite capsules, brought the inspection of a singularly complete armoury of surgeon's requisites to an end.

Messrs. Ferris, of Museum Street, W.C., showed their ingenious artificial limbs. Some time back we described at length these novel arrangements by which a natural movement is given to the artificial limb. So life-like is it, that often spectators have been quite unaware that the wearer had borrowed anything from art to eke out his deficiencies. Messrs. Weiss, of Oxford Street, showed a large number of new and interesting surgical instruments, which quite sustained the reputation of this old and well-known firm.

